# A prospective comparative study to assess the contribution of radioisotope tracer method to dye-only method in the detection of sentinel lymph node in breast cancer

**DOI:** 10.1186/1471-2482-13-13

**Published:** 2013-04-25

**Authors:** Mehmet Eser, Metin Kement, Levent Kaptanoglu, Melin Gecer, Evrim Abamor, Firat Tutal, Salim Balin, Necmi Kurt, Huseyin Uzun

**Affiliations:** 1 Department of General Surgery, Kartal Training and Research Hospital, Istanbul, Turkey; 2 Department of Pathology, Kartal Training and Research Hospital, Istanbul, Turkey; 3 Department of Nuclear Medicine, Kartal Training and Research Hospital, Istanbul, Turkey; 4 Kartal Egitim ve Arastirma Hastanesi Cevizli-Kartal, Istanbul, Turkey

**Keywords:** Breast cancer, Axilla, Sentinel lymph node, Blue-dye, Radionuclide

## Abstract

**Background:**

Metastasis in the axillary lymph nodes is the most important known prognostic factor for breast cancer. We aimed to investigate the contribution of the radioisotope tracer method to the dye-only method by performing sentinel lymph node biopsy on the same patient group during a single surgical session.

**Methods:**

Forty-two patients who underwent operations in our clinic from February 2010 to October 2011 and with masses of <5 cm and clinically and radiologicallly negative axilla (T1-2 N0) were prospectively included in this study. After paraffin examination results were obtained, the numbers and metastatic states of the lymph nodes that were unidentifiable during surgery (although they were stained) but were detected by a gamma probe, lymph nodes that were only stained, lymph nodes that were only radioactive (hot), and lymph nodes that were both stained and radioactive (stained-hot) were determined in all patients. In patients who underwent axillary lymph node dissection, the total numbers of lymph nodes removed and their metastatic states were determined separately.

**Results:**

At least one blue-stained sentinel lymph node was identified in all patients during the blue-stained lymph node detection stage. The average number of sentinel nodes removed at this stage was 2.1 ± 1.1. In the second surgical stage (the stage in which nodes with axillary counts were investigated with the gamma probe) in these 41 patients, at least one additional hot node was removed, or at least one of the nodes that was removed because it was blue was also hot. In addition to the lymph nodes removed in the dye stage, 34 hot lymph nodes were excised from 21 patients. Overall, the average number of hot lymph nodes removed was 2.9 ± 1.5. In all patients, subsequent frozen sections and histopathological examinations were 100% concordant with the sentinel lymph nodes that were removed; the stained sentinel lymph nodes that were removed first did not affect the decision to perform axillary dissection.

**Conclusion:**

The results of our study indicate that performing sentinel lymph node biopsy with dye only is sufficient and as effective as the combined method.

## Background

In breast cancer, the most important known prognostic factor is the presence of metastasis in the axillary lymph nodes [1]. In early-stage breast cancer, knowledge of the presence or absence of axillary involvement can be obtained by axillary lymph node dissection (ALND). However, it can also be obtained with high sensitivity and accuracy by sentinel lymph node biopsy (SLNB), which is a much less invasive method [2-4]. SLNB can be performed using a dye-only method, a radioisotope-only method, or a combination of the two methods. With all three methods, the status of the axilla can reportedly be presented with high sensitivity and accuracy and acceptable false-negative rates [5-7].

Compared with the combination method, performance of SLNB with the dye-only method is an inexpensive and practical technique that does not require involvement of the nuclear medicine department. Randomised studies that compared the combination method with the dye-only method could not adequately demonstrate that the combination method was superior to the dye-only method; this topic is still under debate [5,7-14]. Important studies on this topic have been conducted by applying and comparing the different techniques using different randomised patients. However, comparison of the two techniques on the same patients during the same surgical session will completely equalise patient-derived factors and facilitate the acquisition of more accurate results.

In this study, we aimed to investigate the contribution of the radioisotope tracer method to the dye-only method by performing SLNB on the same patient group during a single surgical session.

## Methods

Forty-two patients who underwent operations in our surgical department (a level three referral hospital) between February 2010 and October 2011 with masses of <5 cm and clinically and radiologically negative axillae (T1-2 N0) were prospectively included in this study. A total of 123 patients underwent operations within this time frame in our institution (56 SLNB and 67 ALND). Fourteen of 56 patients (SLNB) were excluded from our study because they underwent operations by different surgeons. The exclusion criteria were pregnancy, having a history of axillary surgery, having received radiotherapy in the axillary region, having received neoadjuvant chemotherapy, and having allergies to the dye and/or albumin. The age of the patients, their age at menarche, whether or not they were in menopause, the localisation of the tumour, the radiologic Breast Imaging Reporting and Data System (BI-RADS) score, the type of biopsy performed for diagnosis, the type of surgery that took place, the knowledge of the tumour T stage, and the histological results were recorded on a form that was prepared prospectively. The study was approved by Dr. Lutfi Kirdar of the Kartal Training and Research Hospital Ethics Committee. Informed consent forms were obtained from all patients.

In the nuclear medicine clinic of our hospital on the morning of the surgery, 0.2 ml and 20 Mbq (99 m) of Tc-nanocolloid (CIS Bio International, France) was injected around the tumour in all patients. In excisional biopsy patients, it was also subcutaneously injected around the excision region (pouch). Immediately following the injection, the SLN was imaged by performing both a dynamic and static lymphoscintigraphy series. The marking of the skin projection of the SLN with a pen, as is performed in routine applications, was not performed in these patients. Patients underwent operations within 2 hours.

An experienced surgical resident performed a scintillation count with a gamma probe. He compared the results with the lymphoscintigraphy report and recorded the data. He did not share this information with the primary surgeon until the end of the first stage (dye guidance stage) of the surgical procedure. The primary surgeon was informed after the use of patent blue violet dye and performed all surgeries with this knowledge. For all patients, SLNBs were performed by the same surgeon under general anaesthesia. During the surgery, 1% patent blue violet, which was prepared in 5-ml sterile ampoules (Sigma-Aldrich, Germany) in the Istanbul University Faculty of Pharmacy, was used as the dye. The SLNB was performed by injecting one entire ampoule subcutaneously lateral to the incision in patients with a biopsy scar in the upper outer quadrant of the breast and subcutaneously in the subareolar region in the other patients. Following the injection, the dye was gently massaged toward the axilla for 7 minutes in all patients. One centimetre below the axillary line, a small transverse incision was applied and the stained lymph channel, which led to the SLN, was detected. By searching the SLNB, no additional effort was applied to extract the blue channels, but these channels were also removed with the SLN. After the axillary dye guidance procedure was completed and all stained nodes were believed to have been excised, the primary surgeon was informed about the location and number of the foci found by lymphoscintigraphy, the foci detected, and their count values as detected by the hand-held gamma probe in the axilla before the incision. Counts from the excised stained lymph nodes were taken and recorded. The remaining axillary lymph nodes were then scanned with an intra-operative hand-held gamma probe; if there were additional lymph nodes that emitted counts, they were also excised. The lymph nodes were specially examined to determine whether they retained the dye and thus verify whether any stained nodes were missed, the dye properties and count values were recorded, and the nodes were numbered and sent for peri-operative pathological examination. In the pathological examination, SLNs were sectioned into two parts along their long axes by a pathologist experienced in this field. Imprint preparations were prepared from the cross-sections. Preparations were stained with haematoxylin-eosin (H-E). One half of each SLN was reserved for paraffin examination. The other half was examined macroscopically by preparing 2-mm sections, and appropriate sections were flash-frozen. Samples were taken from frozen preparations, stained with H-E, and examined by light microscopy. Two-millimetre sections were prepared from the other half of the lymph node, and paraffin blocks were obtained from each segment after they were fixed in Hollande solution. Serial sections 3 to 4 μm in thickness were prepared from these blocks. Samples were taken and stained with H-E; a standard examination was then performed. Based on the results of the frozen sections (FSs), axillary dissection was performed for the patient if required. Axillary lymph node dissection was not performed on patients whose FS results were negative.

After the results of the paraffin examinations for all patients were obtained, the numbers and metastatic states of the lymph nodes were determined separately for those lymph nodes that were not noted during the dye surgery (although they were stained) but were detected because counts were obtained by the gamma probe, for the lymph nodes that were only stained, for the lymph nodes that were detected with radioactivity only (hot), and for the lymph nodes that both retained the dye and were detected as radioactive (stained-hot). In patients who underwent ALND, the total numbers of lymph nodes removed and their metastatic states were determined separately.

## Results

A total of 42 patients were included in our study. All of the patients were female and their average age was 51.3 ± 11.9 years. The demographic and clinical characteristics of the patients are shown in Table 1. In one patient who underwent direct ALND, no SLN was detected by lymphoscintigraphy, gamma probe, or blue dye, and no metastasis was observed in the axilla of this patient. In the remaining 41 patients, at least one stained-only, hot-only, or stained-hot SLN was found.

**Table 1:** Patient clinical and demographic information

**Mean age**	**51.3 ± 11.9**
Premenopausal	18 (43%)
Postmenopausal	24 (57%)
Tumour localisation	
Central	8 (19%)
Upper outer quadrant	19 (45%)
Lower outer quadrant	3 (7%)
Upper inner quadrant	6 (14%)
Lower inner quadrant	5 (12%)
Mammographic BI-RADS	
BI-RADS 0	7 (17%)
BI-RADS 3	2 (5%)
BI-RADS 4a	2 (5%)
BI-RADS 4b	2 (5%)
BI-RADS 4c	7 (17%)
BI-RADS 5	21 (50%)
Ultrasonography BI-RADS	
BI-RADS 0	1 (2%)
BI-RADS 3	1 (2%)
BI-RADS 4a	3 (7%)
BI-RADS 4b	3 (7%)
BI-RADS 4c	13 (31%)
BI-RADS 5	21 (50%)
Lumpectomy + SLN	19 (45%)
Lumpectomy + AD	12 (29%)
Mastectomy + SLN	3 (7%)
Mastectomy + AD	8 (19%)
Tumour diameter (T)	
T1a	0
T1b	3 (7%)
T1c	12 (29%)
T2	27 (64%)
Tumour histology	
Invasive Ductal	38 (90%)
Invasive Lobular	1 (2%)
Mucinous carcinoma	2 (5%)
Squamous cell carcinoma	1 (2%)

In our study, at least one blue-stained SLN was found in the first surgical stage (the stage in which only the blue-stained lymph nodes were detected). In the first stage, a total of 87 stained nodes were extracted. Seventy-one of these nodes were also found to be hot by ex-vivo counting. In the second stage, in which remnant hot lymph nodes in the axilla were detected with gamma hand probe guidance, a total of 34 hot nodules were removed. Three of these were stained nodes that we could not detect in the first stage in three different patients. These findings did not affect the decision to perform axillary dissection because of same metastatic status of these nodes. A total of 74 stained-hot nodes were extracted from 41 patients. Nineteen patients had 23 metastatic lymph nodes. The ratio of metastatic nodes was 31.08% in the stained-hot node group.

Sixteen stained-only lymph nodes were extracted from eight patients. Four of these eight had a total number of five metastatic lymph nodes. The metastatic status of these nodes was correlated with the metastatic status of the stained-hot lymph nodes in each single patient. The ratio of metastatic nodes was 31.3% in the stained-only group.

In the second stage, 34 hot nodes were removed from 19 patients (3 of these nodes were missed stained-hot nodes that were not found in the first stage). Three metastatic lymph nodes were found in three different patients in this group. The metastatic status of these nodes was correlated with the stained-hot nodes in each single patient. The ratio of metastatic nodes was 8.83% in the hot-only group.

The total number of stained lymph nodes was 90, and the ratio of metastatic nodes was 31.1%. The total number of hot nodes was 108, and the ratio of metastatic nodes was 24.1%.

In the first stage, the average number of SLNs removed was 2.1 ± 1.1. In the second stage of surgery (the stage in which the nodes that gave axillary counts were detected), at least one additional hot node was removed from 41 patients, or at least one of the nodes that was removed in the first stage because it was blue was also hot. With the gamma probe, 34 hot lymph nodes were excised from 21 patients in addition to the lymph nodes that were already removed in the dye stage (median, 1; average, 1.6; range, 1–4). Overall, the average number of hot lymph nodes that were removed was 2.9 ± 1.5. In three patients (7% of all patients) with additional lymph node removals, these single hot lymph nodes were also stained, but were not noted during the first stage of the procedure. In all patients, including these three patients, FS and subsequent histopathological examinations of the SLNs that were removed later and the SLNs that were removed first were 100% concordant and did not affect the decision to perform axillary curettage (Tables 2 and 3). ALND was performed on a total of 20 patients (48%), 19 of whom had positive axillary lymph nodes (46%). As a result of ALND, an average of 19.7 ± 5.6 (range, 10–29) lymph nodes were dissected.

**Table 2:** Patient distribution according to stages

**Data**	**Blue dye**		**Combination method**	
	***n***	***%***	***n***	***%***
Number of patients recruited to the study	42	100	42	100
Number of patients with SLN detected	41	97.6	41	97.6
Number of patients with positive SLN	19	45.2	19	45.2
Number of patients with negative SLN	22	53.7	22	53.7
Number of patients with stained-hot lymph nodes that were missed during the blue dye stage (%)	3 (7.1)			
Number of patients with different frozen section results with the blue dye and combination methods	0 (0)			

**Table 3:** Lymph node distribution according to stages

**Data**	**Blue dye**	**Combination method**
Number of lymph nodes removed	87	121
Average number of lymph nodes removed (SD)	2.1	3.0
Number of stained-hot lymph nodes missed at the blue dye stage (%)	3 (3.2)	
Number of patients with different frozen-section results with the blue dye and combination methods (%)	0 (0)	

## Discussion

The SLN can be defined as the first lymph node that receives the lymphatic drainage of the region containing the primary tumour. SLNB is now a standard technique that is applied to early-stage breast cancer with clinically negative axillae; it can predict the presence of metastasis with a high rate of accuracy [4,15]. Technically, SLNB can be applied using only dye, only radioactivity, or a combination of these two methods. All three methods can reportedly be applied with high rates of detection, sensitivity, and accuracy and with acceptable false-negative rates [10].

Performance of SLNB with blue dye only is a more inexpensive and easier technique to apply compared with the combination method because it does not require involvement of the nuclear medicine department. In studies using these methods, the success rates of finding SLNs are reported to be 41% to 100% with the dye-only method, 69% to 100% with the radioisotope-only method, and 76% to 100% with the combination method [16-18]. Kim et al. investigated 69 clinical studies performed between 1970 and 2003 and calculated a false-negative rate of 10.9% with the dye-only method, 8.8% with the radiocolloid-only method, and 7% with the combination method in their meta-analysis [16]. The relatively low success rates reported in the literature for SLNBs performed with the dye-only method can be explained by the fact that in these studies, the initial SLNB was performed with the dye-only method [7].

When comparing the dye-only and combination methods, Radovanovic et al. reported that in their prospective randomised study with 150 patients, although there was no significant difference in the number of lymph nodes removed, the combination technique was superior in its negative predictive value and overall accuracy rates. In this study, the negative predictive value was 86.9% in the dye-only group and 95.3% in the combination group; the overall accuracy was 68% in the dye-only group and 83% in the combination group [13]. Varghese et al., in a prospective randomised study with 329 patients, reported similar results for the two methods and stressed that in institutions without nuclear medicine capability, SLNB can be performed using the dye-only method. and Meyer-Rochow et al. reported comparable findings in their randomised study with 104 patients [14,19]. Koukouraki et al. found that the SLN detection rate was higher with the combination method in their randomised study with 501 patients, but Kern et al. reported a high concordance between the two methods in their study with 185 patients, and similar numbers of SLN detection ratios were obtained with both techniques [12,20].

Three reviews have been published regarding SLNs: in 2006, Steven et al. reported that all of the methods could be performed with similar SLN detection and false-negative rates when they were performed by experienced surgeons; in 2007, Sato et al. reported that the dye and combination methods returned the same results and that the radioisotope added extra cost and potential radioactivity damage risk; and in 2008, Collins reported that radiopharmaceutical-only SLNB was superior to dye-only SLNB but that only the combination method had the potential to maximise the correct staging [5,6,9].

In all of the above-mentioned studies, SLNB was performed by two surgical teams and, as required by the randomisation, the dye-only group and the combination group comprised different patients. Because of this, the surgeon and patient factors could not be equalised in these studies. Our study design is novel because these two factors were completely equalised by having the same surgeon perform all of the surgeries and by applying both techniques to the same patient group.

In our study, as a result of adding the radioisotope to the blue dye, 34 additional lymph nodes were excised from 42 patients; this number is quite high compared with other studies in the literature. In addition, the average number of lymph nodes that were identified for removal by the combination method was significantly higher than the number of lymph nodes removed by the dye-only method. Despite this high additional lymph node dissection, the lymph nodes first removed from the axilla using the dye-only method, the hot-only nodes that were removed later, and the stained-hot nodes that were not missed during the first stage were 100% concordant in terms of metastasis and did not change the decision to perform axillary curettage.

The mean age was low in our present study because of the limited number of patients. In general, the mean age of the patients who undergo breast cancer surgeries in our institution is higher. Furthermore, in Turkey, the mean age of patients with breast cancer is correlated with world numbers. We believe that the low mean age in our present study was incidental.

SLNB may be difficult in obese patients. In the present study, we did not evaluate body mass indices, which is a limitation.

Although the addition of the radioisotope tracer method increases the number of lymph nodes excised from the axilla, it was not found to be superior to the dye-only method with respect to the SLNB detection rates and determination of axilla positivity; furthermore, it did not change the ALND decision for any of the patients. In this context, when the side effects of the combination method (which are perhaps due to the potentially unnecessary removal of additional lymph nodes) are taken into account, we conclude that SLNB should be performed with the dye-only method. The present study is an original surgical method performed by the same surgeon without the need for a control group. Some authors believe that this method does not bring any additional data to the literature and may result in excessive lymph node dissection.

## Conclusions

We believe that the dye-only method is sufficient for SLNB dissection. Results based on a small selected sample, as in the present study, will not lend to powerful conclusions. We believe that the number of patients in future studies must be increased.

### Consent

All of the patients included in the study were informed about the study in detail, and informed consent forms were obtained. The copies of the written consents are available for review by the Editor-in-Chief of this journal.

## Abbreviations

SLNB: Sentinel lymph node biopsy; ALND: Axillary lymph node dissection; SLN: Sentinel lymph node; H-E: Hematoxylin-eosin; FS: Frozen section; BI-RADS: Breast imaging-reporting and data system.

## Competing interests

The authors declare that they have no competing interests.

## Authors’ contributions

ME, NK, HU, FT and SB were involved in patient care. MK supervised the study and wrote the manuscript. MG performed pathological examinations, EA performed nuclear medicine procedures All authors read and approved the final manuscript.

## Pre-publication history

The pre-publication history for this paper can be accessed here:

http://www.biomedcentral.com/1471-2482/13/13/prepub

## References

[B1] Fisher B Bauer M Wickerham DL Relation of number of positive axillary nodes to the prognosis of patients with primary breast cancer. An NSABP update Cancer 1983 52 1551 1557 10.1002/1097-0142(19831101)52:9<1551::AID-CNCR2820520902>3.0.CO;2-3 6352003

[B2] Krag DN Anderson SJ Julian TB Technical outcomes of sentinel-lymph-node resection and conventional axillary-lymph-node dissection in patients with clinically node-negative breast cancer: results from the NSABP B-32 randomised phase III trial Lancet Oncol 2007 8 881 888 10.1016/S1470-2045(07)70278-4 17851130

[B3] Reitsamer R Peintinger F Prokop E Rettenbacher L Menzel C 200 Sentinel lymph node biopsies without axillary lymph node dissection – no axillary recurrences after a 3-year follow-up Br J Cancer 2004 90 1551 1554 10.1038/sj.bjc.6601765 15083184 PMC2409695

[B4] Lyman GH Giuliano AE Somerfield MR American society of clinical oncology. American society of clinical oncology guideline recommendations for sentinel lymph node biopsy in early-stage breast cancer J Clin Oncol 2005 23 7703 7720 10.1200/JCO.2005.08.001 16157938

[B5] Chen LS Iddings DM Scheri R Bilchik AJ Lymphatic mapping and sentinel node analysis: current concepts and applications CA Cancer J Clin 2006 56 292 309 10.3322/canjclin.56.5.292 17005598

[B6] Sato K Current technical overviews of sentinel lymph node biopsy for breast cancer Breast Cancer 2007 14 354 361 10.2325/jbcs.14.354 17986800

[B7] Giuliano AE Kirgan DM Guenther JM Lymphatic mapping and sentinel lymphadenectomy for breast cancer Ann Surg 1994 220 391 398 10.1097/00000658-199409000-00015 8092905 PMC1234400

[B8] Usmani S Khan HA Abu Huda F Evaluation of the gamma probe-guided sentinel lymph node biopsy and the blue dye technique in the management of breast cancer Hell J Nucl Med 2010 13 30 34 20411168

[B9] Collins C The sentinel node in breast cancer Cancer Imaging 2008 8 1 18 10.1102/1470-7330.2008.0001 18852076 PMC2582497

[B10] Hung WK Chan CM Ying M Chong SF Mak KL Yip AW Randomized clinical trial comparing blue dye with combined dye and isotope for sentinel lymph node biopsy in breast cancer Br J Surg 2005 92 1494 1497 10.1002/bjs.5211 16308853

[B11] Cserni G Rajtár M Boross G Sinkó M Svébis M Baltás B Comparison of vital dye-guided lymphatic mapping and dye plus gamma probe-guided sentinel node biopsy in breast cancer J Am Coll Surg 2002 195 467 475 10.1016/S1072-7515(02)01312-1 12098052

[B12] Kern KA Concordance and validation study of sentinel lymph node biopsy for breast cancer using subareolar injection of blue dye and technetium 99 m sulfur colloid Arch Surg 2011 146 393 394 10.1001/archsurg.2011.28 12375751

[B13] Radovanovic Z Golubovic A Plzak A Stojiljkovic B Radovanovic D Blue dye versus combined blue dye-radioactive tracer technique in detection of sentinel lymph node in breast cancer Eur J Surg Oncol 2004 30 913 917 15498633 10.1016/j.ejso.2004.08.003

[B14] Varghese P Mostafa A Abdel-Rahman AT Methylene blue dye versus combined dye-radioactive tracer technique for sentinel lymph node localisation in early breast cancer Eur J Surg Oncol 2007 33 147 152 10.1016/j.ejso.2006.09.026 17081723

[B15] Breast Cancer in National Comprehensive Cancer Network Clinical Practice Guidelines in Oncology .V.2 2006 http://www.nccn.org

[B16] Kim T Giuliano AE Lyman GH Lymphatic mapping and sentinel lymph node biopsy in early-stage breast carcinoma Cancer 2006 106 4 16 10.1002/cncr.21568 16329134

[B17] Mudun A Sanlı Y Ozmen V Comparison of different injection sites of radionuclide for sentinel lymph node detection in breast cancer: single institution experience Clin Nucl Med 2008 33 262 267 10.1097/RLU.0b013e3181662fc7 18356664

[B18] Asoglu O Ozmen V Karanlik H Kecer M Muzlumznoglu M Igci A Parlak M The role of sentinel lymph node biopsy with blue dye alone in breast cancer patients with excisional biopsy Acta Chir Belg 2005 105 291 296 16018523 10.1080/00015458.2005.11679719

[B19] Meyer-Rochow GY Martin RC Harman CR Sentinel node biopsy in breast cancer: validation study and comparison of blue dye alone with triple modality localization ANZ J Surg 2003 73 815 818 10.1046/j.1445-2197.2003.02783.x 14525573

[B20] Koukouraki S Sanidas E Askoxilakis J Is there any benefit from sentinel lymph node biopsy using the combined radioisotope/dye technique in breast cancer patients with clinically negative axilla? Nucl Med Commun 2009 30 48 53 10.1097/MNM.0b013e3283139302 19020472

